# Supplementary material for: Mapping Tumor Spheroid Mechanics in Dependence of 3D Microenvironment Stiffness and Degradability by Brillouin Microscopy
Source: Cancers (Basel). 2021 Nov 5;13(21):5549. doi: 10.3390/cancers13215549 (PMC8583550; doi:10.3390/cancers13215549)
Supplement: Supplementary file 1 [file cancers-13-05549-s001.zip › cancers-1416852-supplementary.pdf]

Supplementary Materials: Mapping Tumor Spheroid Mechanics in Dependence of 3D Microenvironment Stiffness and Degradability by Brillouin Microscopy

Vaibhav Mahajan, Timon Beck, Paulina Gregorczyk, André Ruland, Simon Alberti, Jochen Guck, Carsten Werner, Raimund Schlüßler and Anna Verena Taubenberger

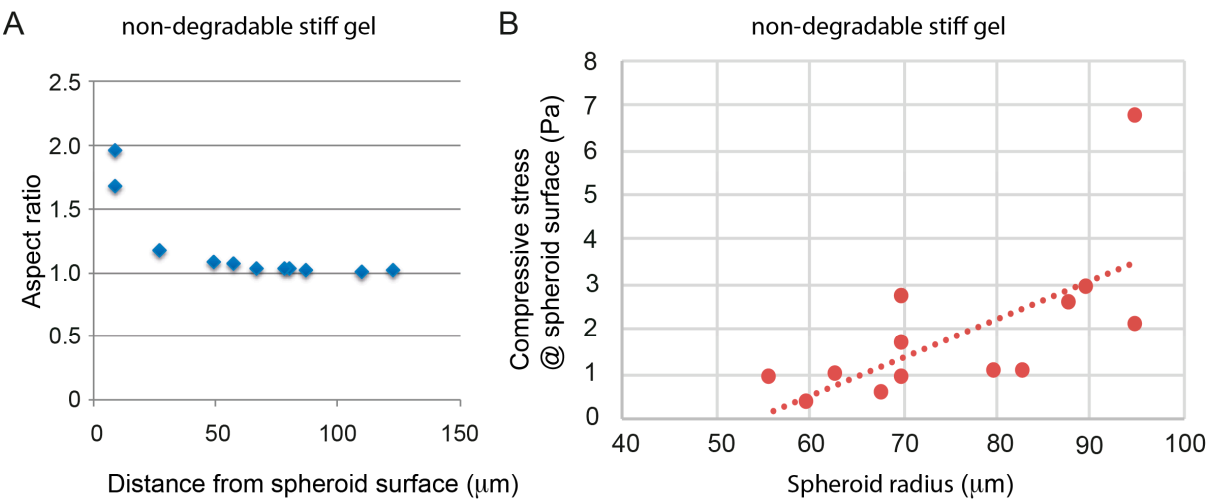

Figure S1. (**A**) Example of bead deformations as determined from confocal images using Fiji. Bead aspect ratios were measured in dependence of the distance from the spheroid surface. (**B**) From maximal aspect ratios corresponding radial stresses were calculated. Stresses were plotted over respective spheroid radii (here for stiff non-degradable hydrogels).

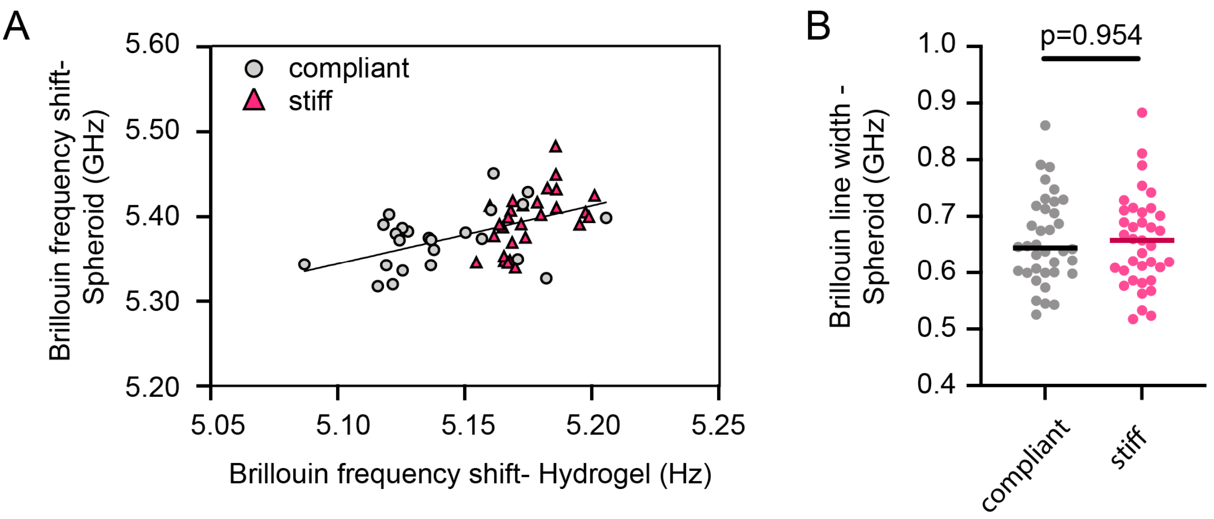

Figure S2. (**A**) Brillouin frequency shifts measured for spheroids over corresponding Brillouin frequency shifts obtained from the surrounding (degradable) hydrogels. A linear trendline is shown. (**B**) Brillouin frequency line width measured for MCF-7 spheroids in compliant and stiff degradable PEG-hep gels. Lines indicate medians. n = 36 (compliant) and n = 37 (stiff). A Mann-Whitney test was performed to compare datasets. p-values are given.

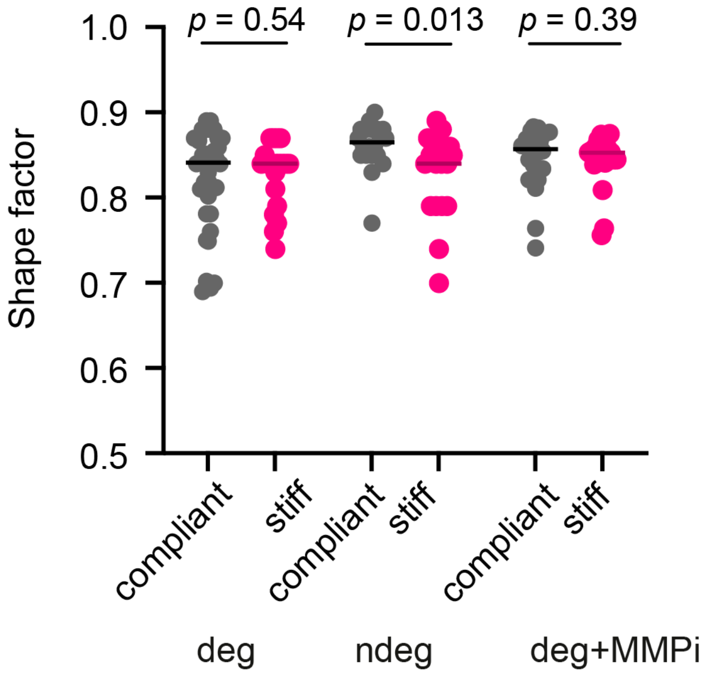

Figure S3. Scatter plots showing shape factors for MCF-7 spheroids formed in compliant and stiff degradable, non-degradable and degradable gels treated with MMPi. Lines indicate medians. n = 10-20 spheroids each. A Mann-Whitney test was done for statistical analysis. p-values are given.

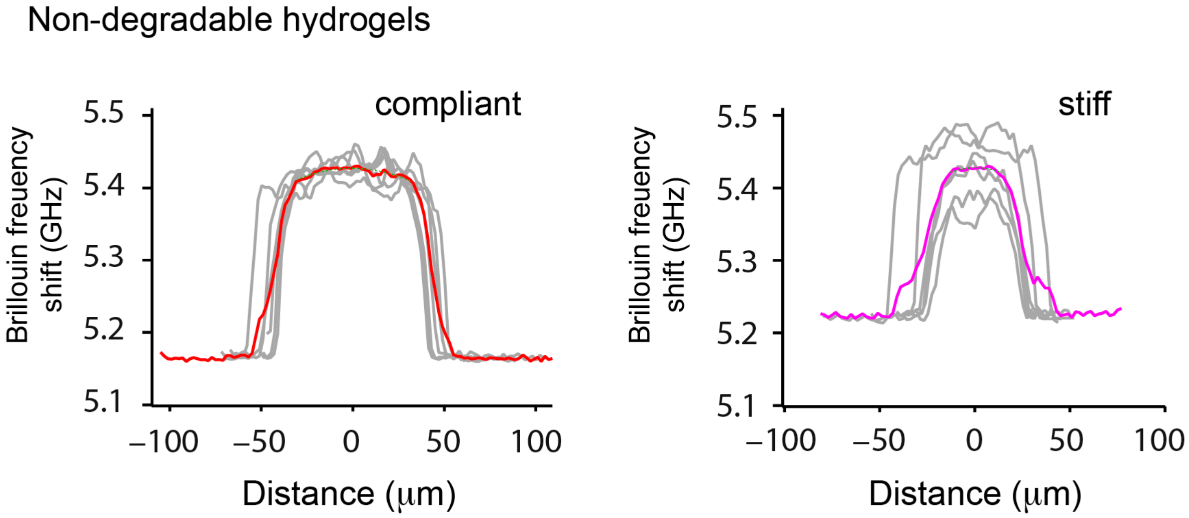

Figure S4. Averaged line profiles of the Brillouin frequency shift across MCF-7 spheroids grown in compliant and stiff non-degradable hydrogels. Red and pink lines show the calculated averages of grey curves for compliant and stiff hydrogels respectively. Analysis was performed using Igor Pro (Wavemetrics).

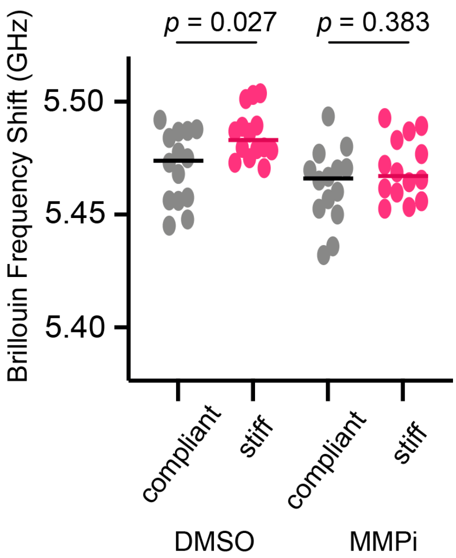

Figure S5. Scatter plots showing Brillouin frequency shifts for MCF-7 spheroids formed in compliant and stiff degradable gels treated with DMSO (vehicle control) and MMPi. Lines indicate medians. $n = 14$ spheroids each. A Mann-Whitney test was done for statistical analysis. p-values are given.

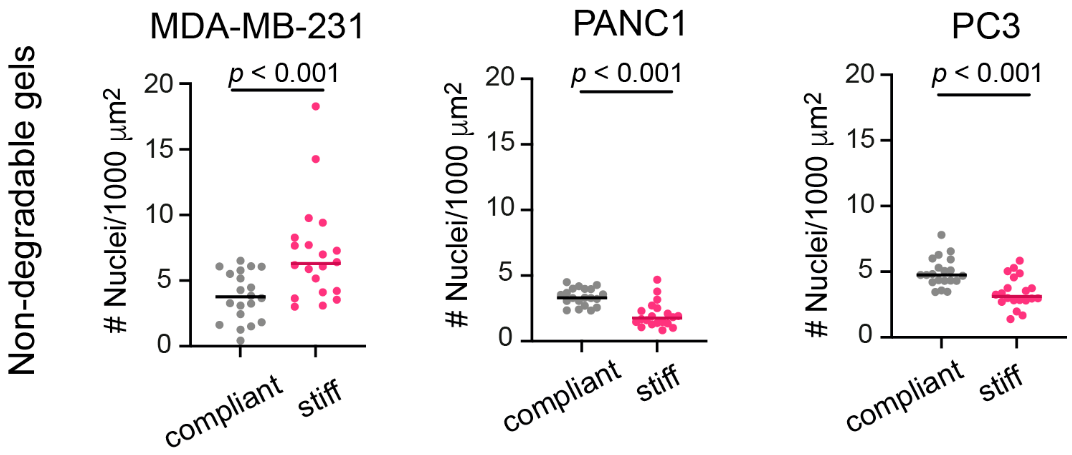

Figure S6. Scatter plots showing number density of cells per unit area of MDA-MB-231, PANC1 and PC3 spheroids formed in compliant and stiff non-degradable gels as determined using FIJI in confocal microscopy images of Phalloidin/DAPI stained spheroid cultures. Lines indicate medians. $n = 20$ spheroids each. A Mann-Whitney test was performed for statistical analysis. p-values are given.

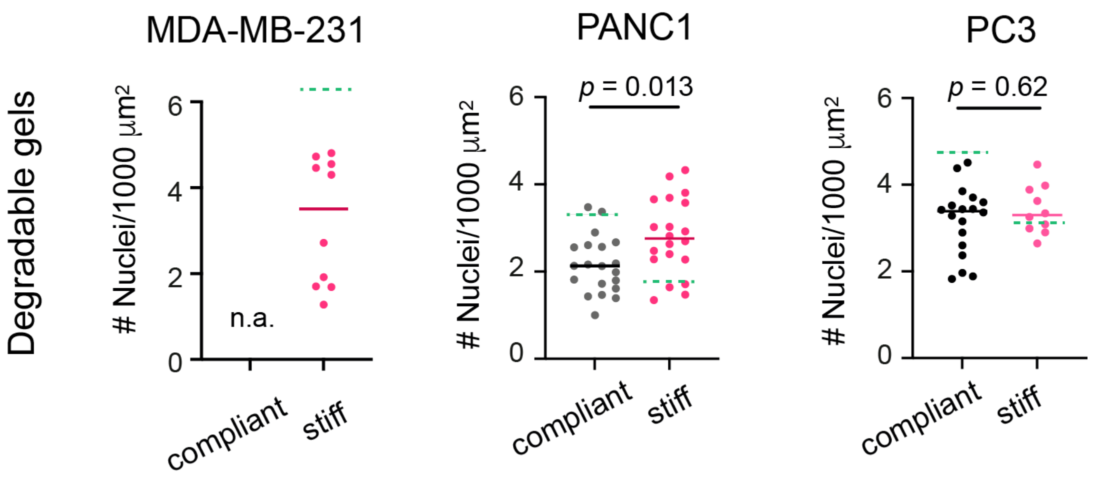

Figure S7. Scatter plots showing number density of cells/nuclei per unit area of MDA-MB-231, PANC1 and PC3 spheroids formed in compliant and stiff degradable gels as determined using FIJI in confocal microscopy images of Phalloidin/DAPI stained spheroid cultures. Lines indicate medians. n = 10-20 spheroids each. A Mann-Whitney test was done for statistical analysis. p-values are given.

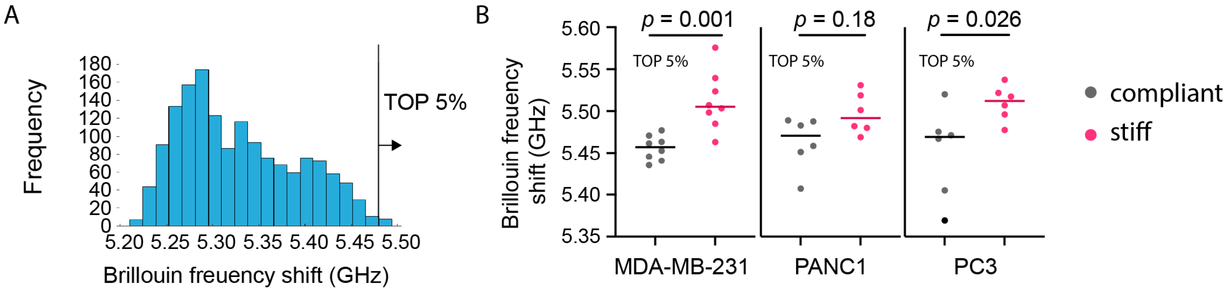

Figure S8. (**A**) Example histogram showing distribution of Brillouin frequency shift values. (**B**) Scatter plots showing the average of top 5% Brillouin frequency shift values of MDA-MB-231, PANC1 and PC3 spheroids formed in compliant and stiff degradable gels. Lines indicate medians. n = 6-8 spheroids each. A Mann-Whitney test was done for statistical analysis. p-values are given.

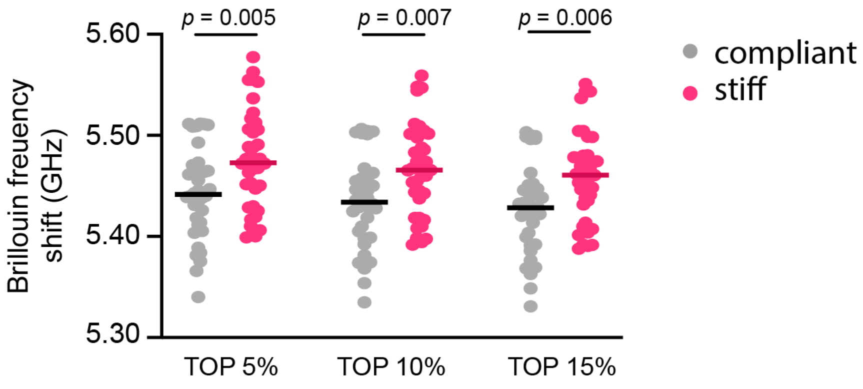

Figure S9. Scatter plots showing the average of top 5%, 10% and 15% Brillouin frequency shift values of MCF-7 spheroids formed in compliant and stiff degradable gels. Lines indicate medians. n = 36-37 spheroids each. A Mann-Whitney test was done for statistical analysis. p-values are given.
